# Predicting clinical scores in Huntington’s disease: a lightweight speech test

**DOI:** 10.1007/s00415-022-11148-1

**Published:** 2022-05-14

**Authors:** Rachid Riad, Marine Lunven, Hadrien Titeux, Xuan-Nga Cao, Jennifer Hamet Bagnou, Laurie Lemoine, Justine Montillot, Agnes Sliwinski, Katia Youssov, Laurent Cleret de Langavant, Emmanuel Dupoux, Anne-Catherine Bachoud-Lévi

**Affiliations:** 1 Département d’Études Cognitives, École Normale Supérieure, PSL University, 75005 Paris, France; 2 Faculté de Médecine, Université Paris-Est Créteil, 94000 Créteil, France; 3 Inserm U955, Institut Mondor de Recherche Biomédicale, Équipe E01 NeuroPsychologie Interventionnelle, 94000 Créteil, France; 4 Centre de Référence Maladie de Huntington, Service de Neurologie, AP-HP, Hôpital Henri Mondor-Albert Chenevier, 51 avenue du Maréchal de Lattre de Tassigny, 94000 Créteil, France; 5 Laboratoire de Sciences Cognitives et Psycholinguistique, CNRS 8554, PSL University, 29 rue d’Ulm, 75005 Paris, France; 6 INRIA, Cognitive Machine Learning Team, 2 Rue Simone IFF, 75012 Paris, France; 7 EHESS, 54 boulevard Raspail, 75006 Paris, France

**Keywords:** Huntington’s disease, Speech, Machine learning

## Abstract

**Objectives:**

Using brief samples of speech recordings, we aimed at predicting, through machine learning, the clinical performance in Huntington’s Disease (HD), an inherited Neurodegenerative disease (NDD).

**Methods:**

We collected and analyzed 126 samples of audio recordings of both forward and backward counting from 103 Huntington’s disease gene carriers [87 manifest and 16 premanifest; mean age 50.6 (SD 11.2), range (27–88) years] from three multicenter prospective studies in France and Belgium (MIG-HD (ClinicalTrials.gov NCT00190450); BIO-HD (ClinicalTrials.gov NCT00190450) and Repair-HD (ClinicalTrials.gov NCT00190450). We pre-registered all of our methods before running any analyses, in order to avoid inflated results. We automatically extracted 60 speech features from blindly annotated samples. We used machine learning models to combine multiple speech features in order to make predictions at individual levels of the clinical markers. We trained machine learning models on 86% of the samples, the remaining 14% constituted the independent test set. We combined speech features with demographics variables (age, sex, CAG repeats, and burden score) to predict cognitive, motor, and functional scores of the Unified Huntington’s disease rating scale. We provided correlation between speech variables and striatal volumes.

**Results:**

Speech features combined with demographics allowed the prediction of the individual cognitive, motor, and functional scores with a relative error from 12.7 to 20.0% which is better than predictions using demographics and genetic information. Both mean and standard deviation of pause durations during backward recitation and clinical scores correlated with striatal atrophy (Spearman 0.6 and 0.5–0.6, respectively).

**Interpretation:**

Brief and examiner-free speech recording and analysis may become in the future an efficient method for remote evaluation of the individual condition in HD and likely in other NDD.

## Introduction

Huntington’s disease (HD) is a rare severe inherited neurodegenerative disease (NDD) whose natural history is well known and well characterized. It combines all complexity of NDDs by associating motor, psychiatric, and cognitive disorders resulting in functional impairment [1]. Despite the development of innovative and promising clinical therapies, a major challenge is the identification of markers sensitive to disease progression, even in the premanifest stage (preHD), before the appearance of motor symptoms.

Current clinical assessments are carried out with the Unified Huntington’s Disease Rating Scale (UHDRS) [2], the worldwide reference scale for HD studies. This is done once or twice a year, during face-to-face examinations performed by trained experts from different specialties (neurologists, neuropsychologists, psychiatrists, and nurses). Each clinical domain is evaluated separately using lengthy, and often subjective scales [3, 4]. Recently, a multi-domain score, named cUHDRS, was proposed as a single endpoint of clinical trials in HD thanks to its greater sensitivity to disease progression [5]. As it combines various scales of the UHDRS, it still requires trained experts and multiple scale assessments. Cognitive batteries with time-dependent tasks [6], brain imaging with striatal volumes [7] or biofluids with Human Cerebrospinal Fluid (CSF) Neurofilament level [8] have also been evaluated as potential markers. These three types of markers have been considered as candidate biomarkers to follow the evolution of HD. However, they all require the presence of the patient at the hospital and a high level of expertise or equipment. In particular (1) cognitive batteries are carried out face-to-face by an expert neurologist/neurologist; (2) high quality brain imaging requires visits of the patient to the neuroimaging center with expensive equipment; (3) analysis of biofluids such as CSF imposes an invasive procedure, which additionally cannot be performed outside hospital under clinical surveillance.

This calls for objective, cost-effective tests to measure the symptoms in a unified approach [9–11]. Neurodegenerative disorders are complex and heterogeneous at the individual level. It is very unlikely that a single marker/measure would have all the good properties for diagnostic and severity assessments of different types of symptoms and truly help for real life clinical decisions. Yet, the combination of complementary biomarkers appears to be a more promising path to predict accurately the different clinical symptoms. Traditional methodologies used in Neurology, Inferential or Bayesian statistics, cannot handle and properly digest very high dimensional data, especially when the number of markers is on par or outnumber the number of data points in the cohort. Making accurate predictions at the individual level becomes possible with machine learning methods. These methods are designed to detect subtle patterns, taking into account a large number of variables, potentially with non-linear interactions [12, 13]. Thanks to increasing computing power, machine learning models now provide an effective methodology to analyze the high-dimensional output of sensors, such as microphones or smartwatches, yielding a patient-tailored approach. This could lead to improved efficiency of the screenings and evaluations of disease modifying therapies by capturing the different clinical dimensions of HD [11].

In this context, speech and language offer an appealing alternative unlocking potential remote evaluation and offering a relevant multi-domain approach. Speaking invokes complex motor abilities [14], cognitive control, and planning at multiple linguistic levels [15]. HD participants are impaired during different steps of spoken language production: phonetics and prosody [16–22], syntax and morphology [23], semantic [24, 25] as well as timings and pauses [26–28], making spoken language a good candidate for clinics. Significant differences were found between healthy controls and HD groups for acoustic markers [16, 27] and language markers [26]. Among these markers, it was found that the speech rate correlates with disease burden score, probability of disease onset, the estimated years to onset, and cognitive score [19, 27]. In addition, speech analysis combined with machine learning models allowed the discrimination of manifest HD and PreHD individuals from controls [29, 30]. However, some of these speech tasks suffer some drawbacks, such as the requirement of fastidious annotation by linguistic experts or language adaptation difficulties, which make their use not suitable for clinical practice; and their sensitivity to the various HD symptoms remain unknown [31].

To fill this gap, we test the capacity of speech to predict the main clinical variables of the UHDRS (cUHDRS, motor, functional, and cognitive) in carriers of the mutant Htt gene. Participants performed a quick speech test consisting of counting forward and backward numbers. We developed a method to quantify articulation, rhythm, perseveration, and vocalization additions. Machine learning models were trained and assessed on different sets of participants to ensure generalization of our results. Finally, the clinical value of speech features was further substantiated by their correlations with the striatal atrophy, the anatomical hallmark of HD [1].

## Methods

**Fig. 1:**
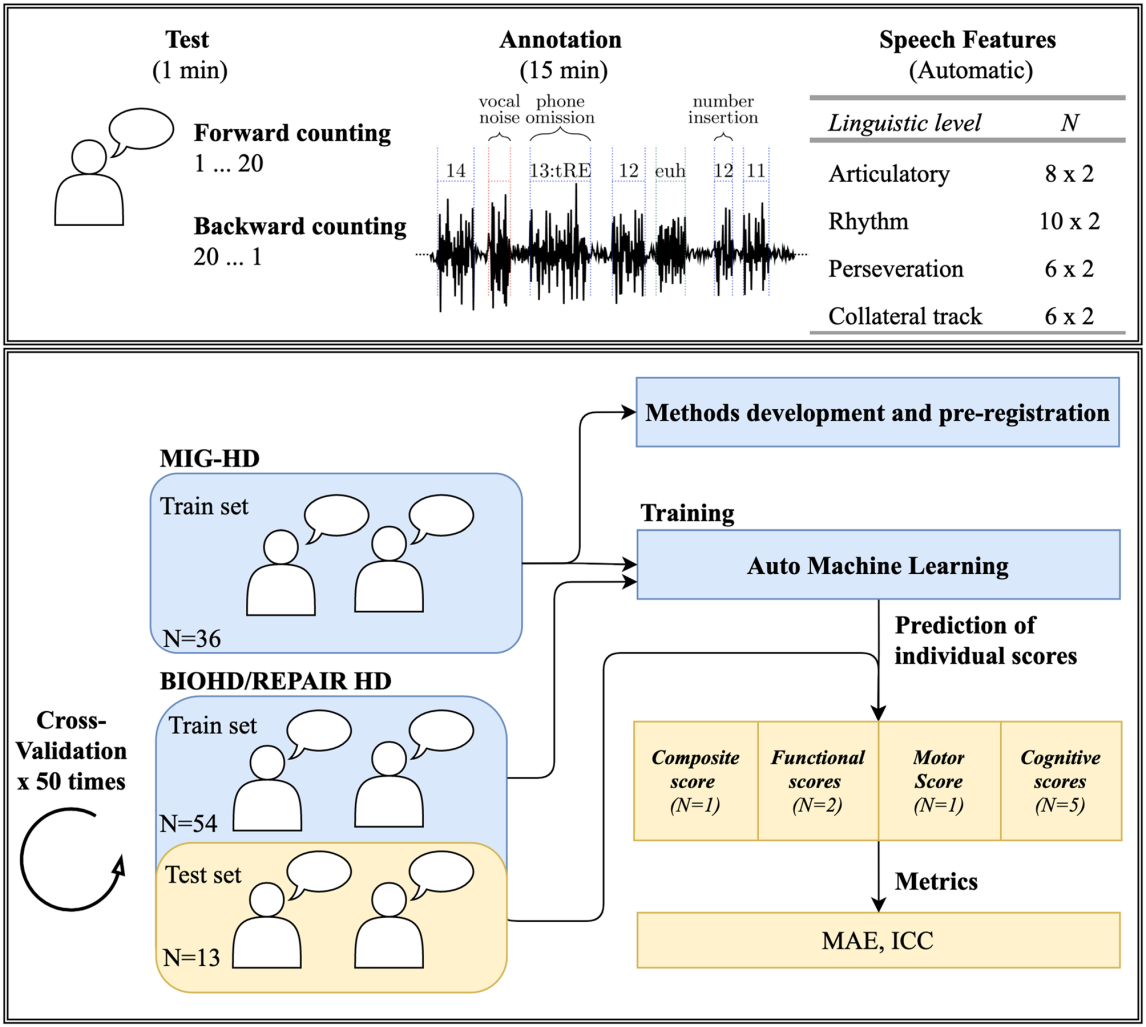
Extraction of individual clinical scores from the speech samples. (Top panel) Examples of portions of the speech signal and various types of vocalizations and segmentation are provided. Similar speech features were extracted separately from the forward and backward counting tasks yielding to 60 features (30 × 2). (Bottom panel) Illustration of the methods developments, Machine learning training and evaluation of the predictions of the clinical scores. *N CAG* number of CAG repeats on the Huntingtin gene, *DBS* Disease Burden Score.* TFC* Total Functional capacity, *TMS* Total motor score, *SDMT* Symbol digit modality, *UHDRS IS* UHDRS Independence Scale, *MAE* Mean absolute error, *ICC* Intraclass correlation coefficient, *cUHDRS* composite UHDRS

We pre-registered all the methods before running the analyses to ensure its reliability and avoid inflated results (https://aspredicted.org/blind.php?x=/66K_66C). We developed the methods with a first cohort (the Multicentric intracerebral grafting cohort, MIG-HD, NCT00190450) and then pre-registered. This first cohort is only used for training models, but the validation was only performed with independent cohorts (see Fig. 1).

### Participants

French native speakers (*N* = 103) individuals with at least 36 CAG repeats on the mutant Htt gene of HD were included in this study (Table 1). One visit refers to one visit to the hospital for a given participant. All assessments were performed on the same visit. Participants were enrolled from three prospective studies: 36 manifest HD from MIG-HD prior to any intervention in 6 centers in France and Belgium from Stage I to Stage III, as defined by the Total functional capacity (TFC)[32], and 67 (51 manifest and 16 PreHD) from both the BIOHD (NCT01412125) and Repair-HD (NCT03119246) cohorts. PreHD participants were defined by a TFC score at 13 and a total motor score (TMS) of the UHDRS equal or below five [33]. The Disease Burden Score (DBS) was computed using the formulae: ${\text{age}} \times \left( {{\text{CAG}}\;{\text{length}} - 35.5} \right)$ [33]. All participants signed an informed consent. Ethical approval was given by the institutional review board from Henri Mondor Hospital (Créteil, France) for the French part of MIG-HD, Bio HD and Repair-HD, and the institutional review board from Erasme Hospital in Belgium. It complied with the Helsinki Declaration, current Good Clinical Practice guidelines, and local laws and regulations.

**Table 1:** Demographics and clinical performance of the participants in the cohorts under study at baseline

	MIG-HD	BIOHD/REPAIRHD	Total
Number of participants	36	67	103
Premanifest/manifest	0/36	16/51	16/87
Number of visits per patient	1.4 (0.5) [1–2]	1.1 (0.3) [1–2]	1.2 (0.4) [1–2]
Gender	23F/13 M	40F/27 M	63F/40 M
Age at first visit	47.0 (9.1) [28–68]	52.7 (11.8) [27–88]	50.7 (11.2) [27–88]
Laterality	30R/5L/1A	59R/8L/0A	89R/13L/1A
Number of CAG repeats	45.3 (4.4) [37–60]	43.5 (3.1) [39–55]	44.0 (3.6) [37–60]
cUHDRS mean (SD) [range]	9.1 (2.5) [5.2–15.0]	11.1 (4.6) [2.5–18.8]	10.4 (4.0) [2.5–18.8]
Total motor score mean (SD) [range]	35.0 (13.6) [7–63]	26.7 (20.3) [0–60]	29.6 (18.6) [0–63]
TFC mean (SD) [range]	10.4 (1.7) [6–13]	11.0 (2.2) [5–13]	10.8 (2.0) [5–13]
UHDRS independence scale mean (SD) [range]	85.7 (8.5) [70–100]	88.9 (12.9) [60–100]	87.8 (11.8) [60–100]
Verbal fluency 1 min mean (SD) [range]	28.2 (8.5) [9–45]	27.6 (13.3) [9–62]	27.8 (11.8) [9–62]
Symbol digit modality test mean (SD) [range]	24.8 (7.6) [11–42]	31.9 (15.2) [3–67]	29.4 (13.4) [3–67]
Stroop word mean (SD) [range]	61.9 (15.0) [39–99]	70.7 (24.7) [23–117]	67.6 (22.1) [23–117]
Stroop color mean (SD) [range]	46.6 (11.9) [24–76]	52.3 (18.5) [16–89]	50.3 (16.7) [16–89]
Stroop interference mean (SD) [range]	26.7 (8.8) [11–45]	29.9 (12.8) [7–58]	28.8 (11.6) [7–58]

Mean, (Standard Deviations) [range]

*F* Female, *M* Male, *R* Right, *L* Left, *A* Ambidexter, *TFC* Total Functional Capacity

### Clinical evaluation

Participants were assessed by certified examiners through nine measures classically used for both clinical practice and trial (Fig. 1): the UHDRS Total Motor Score (TMS), five cognitive assessments (the Symbol Digit Modalities Test (SDMT), the Verbal Fluency test 1-min (VF), and the three components of the Stroop test (word (SW); color (SC); interference (SI)), and two functional scales (the Total Functional Capacity (TFC) and the UHDRS Independence scale (UHDRS IS)). We also computed the composite cUHDRS [5] $\left( {{\text{cUHDRS}} = \left[ {\left( {\frac{{{\text{TFC}} - 10.4}}{1.9}} \right) - \left( {\frac{{{\text{TMS}} - 29.7}}{14.9}} \right) + \left( {\frac{{{\text{SDMT}} - 28.4}}{11.3}} \right) + \left( {\frac{{{\text{SW}} - 66.1}}{20.1}} \right)} \right] + 10} \right)$.

### Standardised lightweight speech test

Speech samples were recorded through two brief controlled tasks by the examiner, who provided the instructions to the participants. Each participant was asked to (1) count aloud numbers from 1 to 20 (forward counting), then (2) to count the numbers backwards from 20 to 1 while holding his/her hands up and closing his/her eyes (backward counting). The rationales for these two subsequent tasks are: (1) we wanted to obtain a baseline performance for counting numbers with minimal cognitive load, (2) we wanted to measure performance of HD as cognitive load is higher, due to the inhibition of forward counting and dual tasking. Recording was performed either by video tape, microphone of the computer, or external microphone.

### Speech features

Only samples without too much acoustic noise, as perceptually determined blindly by two speech therapists before data delivery were retained. Thirty five files were discarded in total (33 from MIG-HD, 2 from BIOHD/REPAIRHD). This yielded the analysis of 126 samples, from 103 patients. In the case of a second visit for a participant, this visit can be separated between 1 and 36 months after the first visit. Then, the two speech therapists blindly transcribed each sample at the word level; and when there was a mispronunciation, the word was transcribed at the phonetic level with the Speech Assessment Methods Phonetic Alphabet using the software Praat [34] and the Seshat platform [35]. This is based on the listening of the acoustic signal, and also visualisation of the acoustic signal along the spectrogram. They identified paraphasias, phone perseverations, abnormal breathing, vocal noises, filled pauses (“euh”, “um”), blocks, and prolongations (Table 2). Paraphasias, phone perseverations, blocks, and prolongations are pooled together to count as “pronunciation error”. Abnormal breathing, vocal noises, and filled pauses are considered to play an important part in communication and are referred to as collateral track additions [36].

**Table 2:** List of speech and language features extracted from the recitation of numbers

Dimension	Speech/language feature
Articulatory and phonatory deficiencies	Total number of pronunciations errors
	Ratio of pronunciation errors
	Pronunciation error per second
	Mean intelligibility based on non-intrusive normed speech-to-reverberation modulation energy ratio metric [40]
	SD of the fundamental frequency
	Range of the fundamental frequency
	SD of normalized intensity of vocalizations
	Normalized range of intensity of vocalizations
Rhythm and temporal statistics	Task duration
	Temporal rate of the pronounced numbers
	Mean duration of pronounced numbers
	Pronounced numbers per second
	SD of the duration of pronounced numbers
	Phones per second
	TR of the silences
	Mean duration of silences
	SD of the duration of silences
	Total number of silences
Sequence errors and perseverations	Levenshtein distance between the pronounced numbers and the target sequence (1, 2, …, 19, 20)
	Gestalt similarity between the pronounced numbers and the target sequence (1, 2, …, 19, 20)
	Levenshtein distance between the pronounced phones and the target sequence (phones of 1, phones of 2, …, phones of 19, phones of 20)
	Gestalt similarity between the pronounced phones and the target sequence (phones of 1, phones of 2, …, phones of 19, phones of 20)
	Total number of pronounced numbers
	Total number of pronounced phones
Collateral track additions	Total number of involuntary/abnormal vocalizations
	Involuntary/Abnormal vocalizations per second
	Temporal rate of the involuntary/abnormal vocalizations
	Total number of filled pauses
	Filled pauses per second
	Temporal rate of the filled pauses

SD stands for standard deviation, Temporal rate is defined as the ratio of the total time of a specific class on the total time to perform the task

Time and categorizations of events differences between raters were systematically discussed until agreement between both annotators. Phones were then force-aligned using Hidden Markov models combined with Gaussian Mixture models based on the Kaldi toolkit [37]. An automatic pipeline algorithm was developed to extract the speech features previously selected on previous analyses of the MIG-HD, the exploration cohort. After exploration on MIG-HD, we preregistered all the methodologies before running the analyses on the BIOHD/REPAIRHD cohort.

Based on these annotations, the forced-alignment and the acoustic waveform, we extracted different speech deficits dimension already reported in HD: articulatory and phonatory deficiencies [16, 17, 27, 38], rhythm and temporal statistics [26, 39], filled pauses and vocalizations additions [26, 27, 29], sequence (the order of numbers), and perseveration errors (introduced here for measuring target sequence errors). In total, we examined 60 features that do not need to be adapted to a specific language (See Table 2 for the full detailed list of speech features).

### Machine learning

We used the auto-machine-learning system, auto-sklearn [41] to predict the clinical variables from the speech features. Auto-sklearn uses Bayesian optimization algorithms to find the model with the best cross-validated performance on the training set. The model selection process is performed independently for each clinical score, yielding different predictors and models. We ran and compared three automatic machine learning pipelines by using different sets of inputs:

1. The speech features (Table 2) with the Demographic variables (Gender, Age, Number of CAG repeats, and Disease Burden Score). In machine learning experiments the relationship between the features and target variable is not always linear. Sometimes the relationship between dependent and independent variables is more complex such as polynomial transformation. That is why we used the combination of the Disease Burden Score alongside the Age and Number of CAG repeats.
2. Demographics variables alone, which allow predicting disease’s onset and progression in HD (Gender, Age, Number of CAG repeats, and Disease Burden Score), and represent an important baseline to be compared to [42].
3. The mean baseline performance of each clinical score on the training set (called Cohort Mean Performance in the following sections), which represents the average performance of individuals in the training set. This Cohort Mean Performance is equivalent to what is usually performed with classic statistical methodologies when there is a will to replicate results across cohorts in medicine.

For the auto-machine learning approach, we followed the approach described in detail in the auto-sklearn article [41]. For the auto-machine learning approach, we set a 2-min time limit for each model training for each clinical score as defined by the auto-sklearn toolkit. Each training is limited to 30 s. We used 24 parallel processes for each clinical score and each model. Thus, the minimum number of models tested was therefore 96 models. Then all the best 50 models found on training data during this search are combined (through ensemble strategy).

To assess the respective importance of each speech feature to predict each clinical score, we used a linear regression model with an ElasticNet regularization (Fig. 5). We also ran an ablation study to evaluate the contributions of the backward and forward speech features. An ablation study is a term from the machine learning literature to refer to an experiment to evaluate contributions of specific features. This means that we run the same machine learning analyses based on the subset of features extracted of the forward counting, and on the subset of features extracted on the backward counting, to evaluate contributions of each.

### Validation of models

We used both the Mean Absolute Error (MAE) and the intraclass correlation coefficient (ICC) between the predicted and the observed scores provided by the clinicians. The ICC measures how much the predicted clinical score outputted by the Machine Learning model resembles the observed score. ICC values were calculated using a two‐way random model with absolute agreement. The use of ICC allows comparing the machine learning model to the interrater reliability of clinicians. The MAE quantifies the absolute errors between the observed clinical scores and the predicted scores.

Concerning the sample size of the current study, we wanted enough visits to train the models and enough visits to test the models. The problem of sample size and model validation for machine learning applied in Neurology and Psychiatry has been extensively studied with simulation in these studies [43, 44]. As underlined by the authors, "leave-one-out" strategy leads to unstable and biased estimates of the true performance of a model, and repeated random splits method should be preferred. 20% should be left out for the test set.

Thus, we splitted the data into two sets: “train set” (86% of the participants, i.e. 89 participants, including all participants of MIG-HD and 80% of the ones of RepairHD/BIOHD) for fitting and developing the various models and an independent “test set” (14% of the participants, i.e. 14 participants, consisting in the 20% remaining participants of RepairHD/BIOHD) for model evaluations. We conducted 50 repeated learning-tests to obtain reliable estimates of the performances. There was no overlap between participants of the training and of the test sets to ensure the generalisation of the results. Multiple visits of the same patients were assigned either to the training set, either to the test set to ensure independence.

In addition, the number of samples should be at least 100 to obtain less than 10% of variance on the test score based on the simulation [43, 44]. We had 103 participants and 126 visits in total in this study, which fulfilled all these requirements.

Identifying Significant Relationships with the Striatum.

The association between each of the 60 speech features and the striatal volumes was assessed in thirty-six participants from the BIOHD/REPAIRHD cohorts (23 females, mean age: 52.98 ± 12.56). High-resolution brain MRI scans were obtained on a Siemens Skyra including T1 3D anatomical MP-RAGE images (repetition time: 2300 ms; echo time: 2900 ms; inversion time: 900 ms; flip angle: 9°; acquisition matrix: 256 × 240; slice thickness: 1.2 mm, no inter-slice gap, 176 sagittal sections). We used the FreeSurfer software (https://surfer.nmr.mgh.harvard.edu/) [45] for extracting subcortical volumes. Percentage of striatal volume relative to the estimated intracranial volume was obtained from the caudate nucleus, ventral striatum, and putamen volumes.

When number of associations to be tested is large with limited data, the assessment of significance of variables must consider that: (1) Measures of relationships need to yield a good probability of making a correct decision when assessing significance (power property), (2) the capability to measure the strength of any relationship (linear or not) at a given noise level (equitability property) and (3) the multi-comparison issue. We therefore used the mutual information-based estimators procedure, including the Total Information Coefficient estimator (TICe) and the Maximal Information Coefficient estimator (MICe) [46] to identify and measure the strengths of their relationships [47]. The TICe allows the screening of variables because of its high power, but low equitability and the MICe estimates the strengths of the relationships because of its high equitability but lower power. In addition, speech variables and clinical scores correlations were corrected for multiple comparisons with the Maximum Statistic correction to take into account the correlations between the variables [48].

## Results

**Fig. 2:**
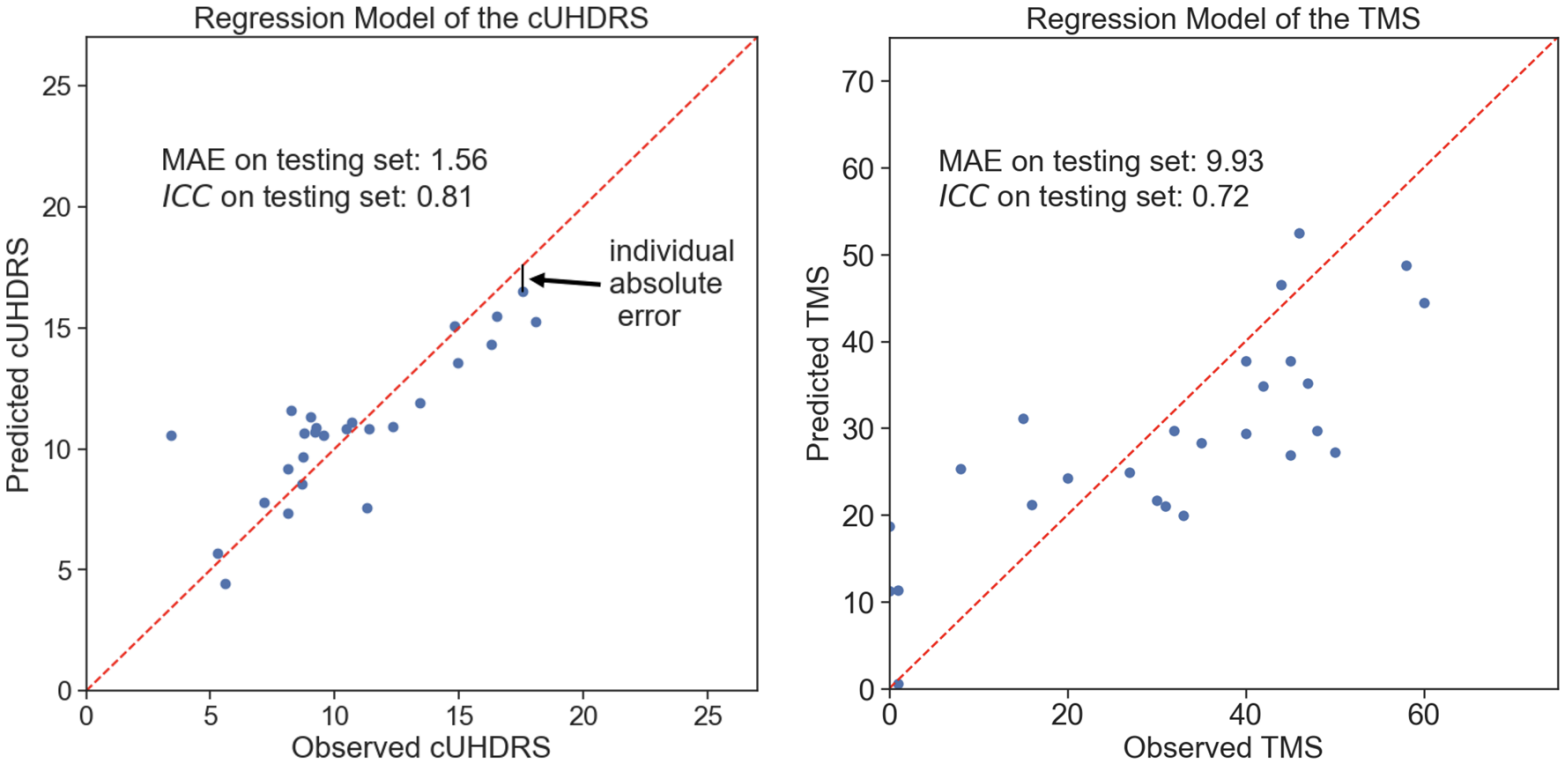
Illustration of individual predictions of the cUHDRS (Left) and the TMS (Right) based on the speech features. Each individual blue dot is the difference between the predicted and the observed score for a particular assessment of an individual of the test set. The red dashed line is the line ‘*y* = *x*’. The black line is the individual contribution of a point (individual absolute error) to obtain the Mean Absolute Error (MAE)

**Fig. 3:**
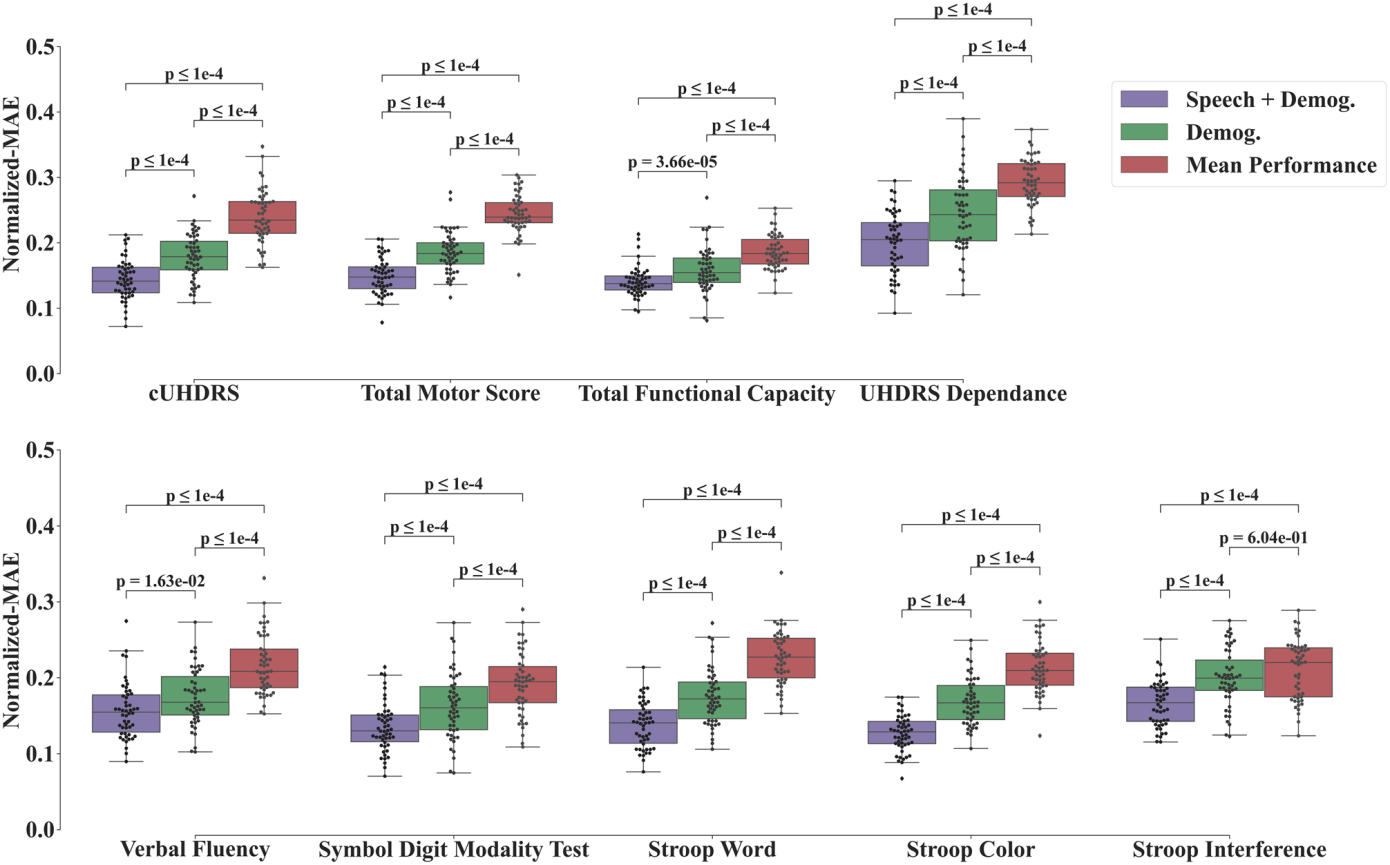
Boxplots of mean-absolute-error (MAE) on the test set for the repeated-learning testing experiment. A MAE at zero means that the predicted value equals the observed one. Horizontal lines are the medians, boxes are upper and lower quartiles, and whiskers are 1.5 × IQR (Interquartile Range). First row displays the cUHDRS, functional, and motor predicted scores; whereas the second row displays the predicted Cognitive Scores. Statistical Significance was assessed with Wilcoxon-test and was Bonferroni-corrected

**Fig. 4:**
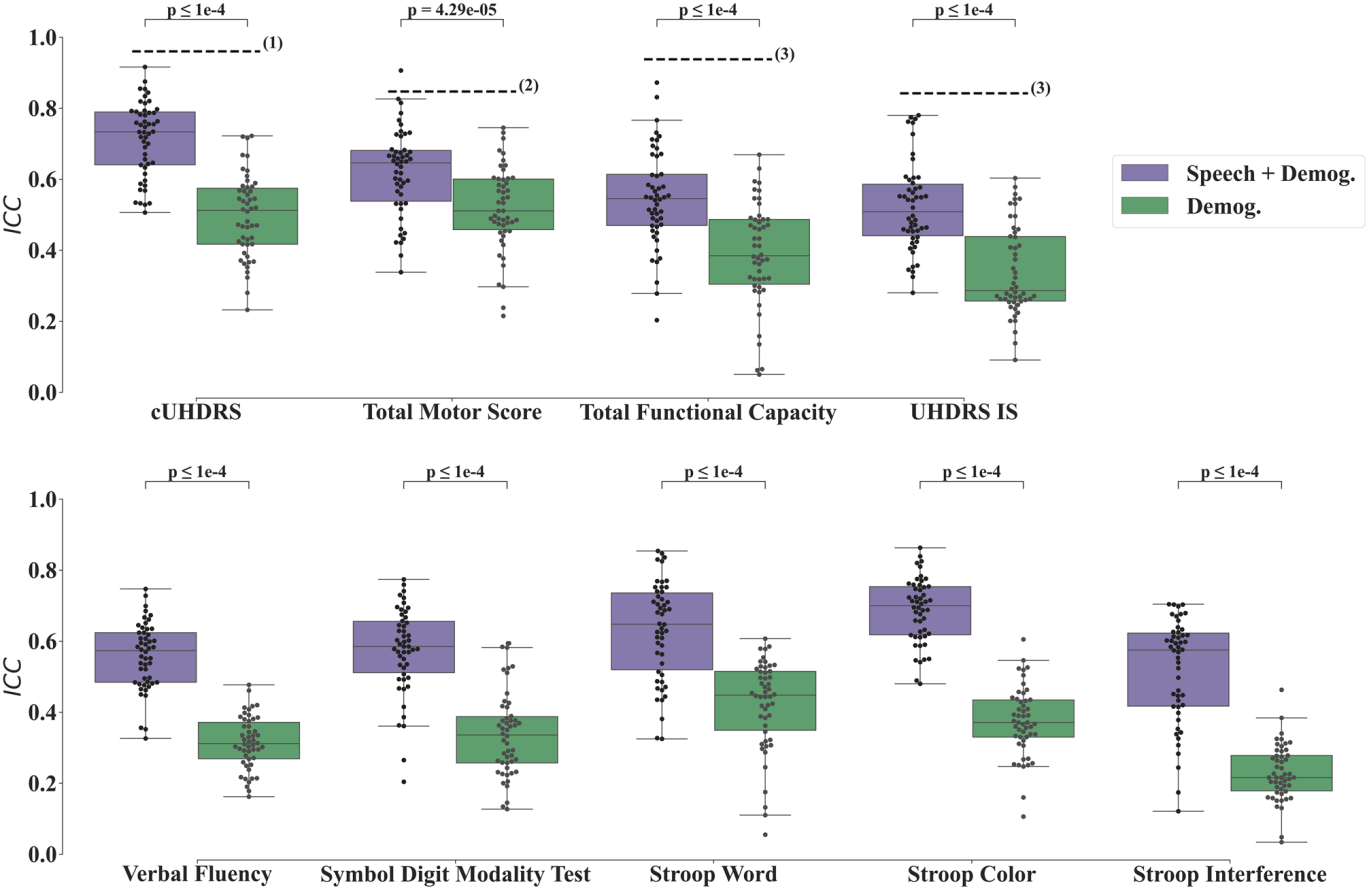
Boxplots of intraclass correlation coefficients (ICC) on the test set for the repeated-learning testing experiment. An ICC at 1 means that the predicted value equals the observed one. Horizontal lines are the medians, boxes are upper and lower quartiles, and whiskers are 1.5 × IQR (Interquartile Range). First row displays the cUHDRS, functional, and motor predicted scores; whereas the second row displays the predicted Cognitive Scores. Statistical Significance was assessed with Wilcoxon-test and was Bonferroni-corrected. The dashed lines figure the ICCs obtained between Neurologists for the clinical scores namely: (1) ICC for cUHDRS ICC = 0.92 [49], (2) for TMS ICC = 0.847 [3], (3) for TFC ICC = 0.938, and for UHDRS IS ICC = 0.842 [4]. The ICC cannot be computed for the Mean Cohort Performance as its standard deviation is zero

The duration for the forward (backward) recitation of numbers is 10.7 $\pm$ 3.6 (15.6 $\pm$ 5.6) seconds. The annotation lasted less than 15 min per file. Illustration of prediction performances of the cUHDRS and TMS are shown in Fig. 2; where each individual prediction error on one visit contributes to the MAE. Predicted clinical scores on the Test Set are displayed in Fig. 3 using the MAE metric and Fig. 4 using the ICC. Models based on the Speech features performed significantly better for the MAE, for all clinical variables, than the ones using the Demographics variables (Age, Gender, Numbers of CAG, and Disease Burden Score) or the Cohort Mean Performance (all *P* values < 0.0001 except for the Verbal Fluency *P* value = 3.25 × 10^−3^, Fig. 3). Models using the Demographics variables performed more accurately than the ones using the Cohort Mean Performance, (all *P* values < 0.0001 except for the Stroop Interference *P* value = 1.32 × 10^−1^, Fig. 3). Models based on the Speech variables performed significantly better for the ICC for all clinical variables, than the ones using the Demographics variables (all *P* values < 0.0001, Fig. 4). Among all variables the cUHDRS was the best predicted based on the ICC. This score is predicted with on average 2.3 points error using the combination of the speech features and demographics (MAE = 2.3 $\pm$ 0.5;$ICC$=0.72 $\pm$ 0.10). Speech and demographic features allowed 19.4% and 29.2% improvement over demographics alone for MAE and ICC respectively, and 40.1% over Cohort Mean Performance models for MAE.

An ablation study showed that the speech features from the backward counting obtain better results overall than the forward ones, and even better results than when combined with the forward ones. Forward speech features obtained for the different scores: cUHDRS MAE = 2.6 $\pm$ 0.5; TMS MAE = 11.7 $\pm$ 1.8; TFC MAE = 1.5 $\pm$ 0.2; UHDRS IS MAE = 8.8 $\pm$ 1.2; VF MAE = 9.2 $\pm$ 1.3, SDMT MAE = 9.8 $\pm$ 1.8; SW MAE = 14.9 $\pm$ 3.1; SC MAE = 10.9 $\pm$ 1.8; SI MAE = 8.9 $\pm$ 1.7. The backward speech features obtained for the different scores: cUHDRS MAE = 2.4 $\pm$ 0.4; TMS MAE = 12.0 $\pm$ 1.8; TFC MAE = 1.3 $\pm$ 0.2; UHDRS IS MAE = 8.1 $\pm$ 1.2; VF MAE = 8.0 $\pm$ 1.0, SDMT MAE = 8.9 $\pm$ 1.8; SW MAE = 13.3 $\pm$ 2.2; SC MAE = 9.6 $\pm$ 1.7; SI MAE = 7.8 $\pm$ 1.5.

Some clinical variables (cUHDRS, TMS, SW, SDMT, and UHDRS IS) and speech features (both Mean duration and Standard Deviation of durations of Silences during backward recitation) correlated with the measure of the striatal atrophy (Table 3). Comparison correction was performed with the Maximum Statistic [48]. The Mean duration of Silences obtained the strongest strength of relationship based on the $MICe$, while the cUHDRS obtained the strongest linear relationship with the Pearson coefficient $R$.

**Table 3:** Summary of the speech and clinical variables with significant correlation with the Normalized Volume of the Striatum

	$TICe$ *P* value	$MICe$	Pearson $R$	Spearman $\rho$
Speech				
Mean duration of the silences during backward recitation	0.0024	0.57	− 0.35	− 0.56
Standard deviation of the duration of the silences during backward recitation	0.026	0.49	− 0.41	− 0.60
Clinical variables				
cUHDRS	0.0050	0.40	0.65	0.68
UHDRS total motor score	0.0090	0.38	0.52	0.57
Stroop word	0.021	0.38	0.61	0.64
Symbol digit modality test	0.030	0.36	− 0.63	− 0.63
UHDRS independence scale	0.040	0.33	0.58	0.57

The comparison between the $TICe$’s *P* values [46], the measure of linear relationship with the Pearson $R$ coefficient, the Spearman rank correlation coefficient $\rho$, the measure of strength of the relationship with the $MICe$ shows that Mean duration of Silences and the Standard Deviation of the duration of Silences are as well correlated with the striatal volume than the regular clinical scores. Multiple Comparison correction is done with the Maximum Statistic [48]

**Fig. 5:**
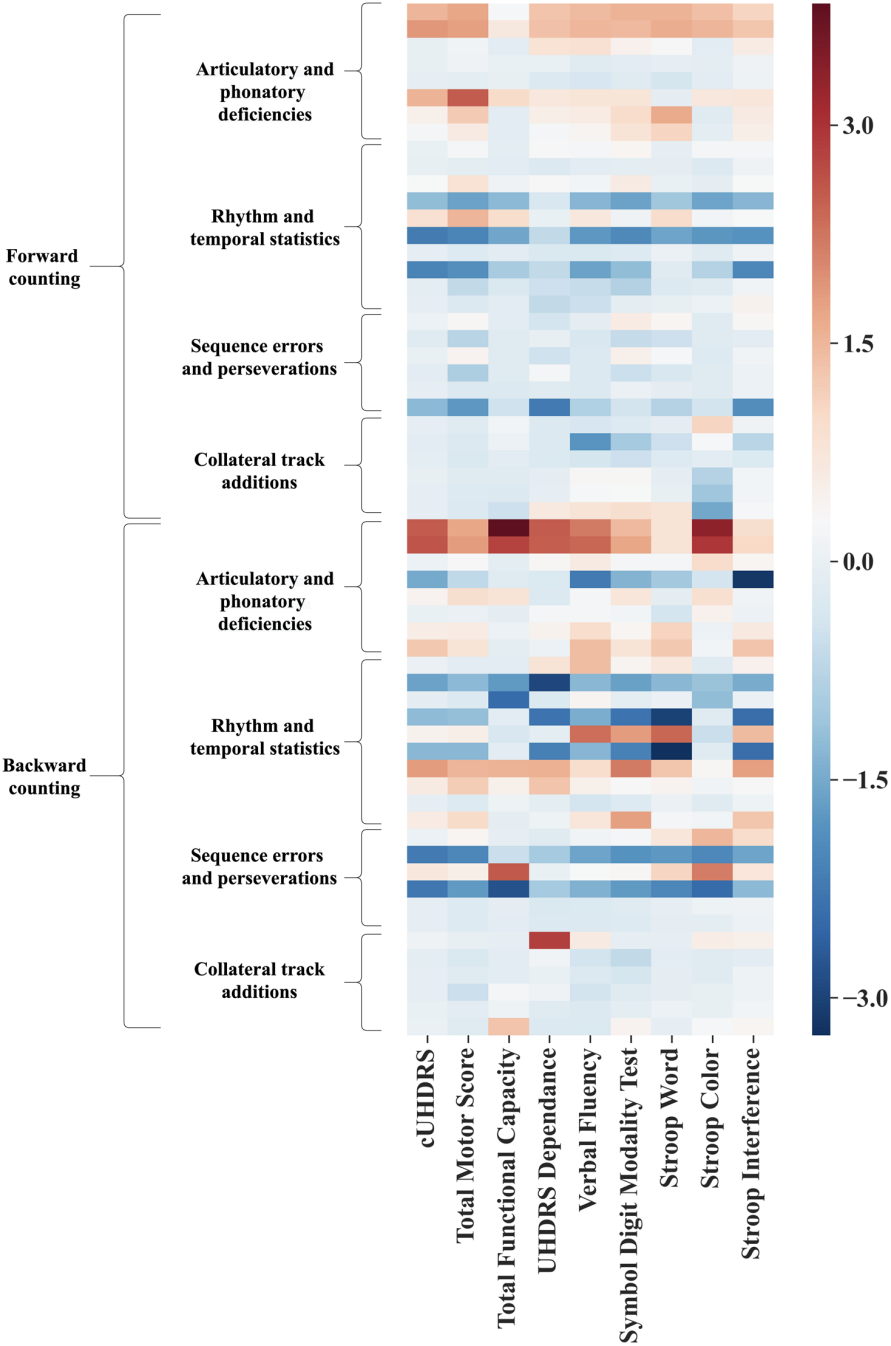
Coefficient importance of the different speech features for the predictions of the clinical scores. Each line represents a feature of Table 2 and the rank is the order introduced in Table 2. These mean weights are obtained with a linear Elastic Net model for interpretability. The weights are z-scored per clinical score to be one the same scale. The weights for the clinical scores are reversed, so that a higher feature weight can be interpreted as a higher clinical impairment

The features that are the most used for predictions are the ones from backward counting (Fig. 5). Speech features extracted from the collateral track additions were less used overall than the other dimensions. Rhythm and temporal statistics were useful for both counting forward and backward.

Even if some coefficients have been set to 0, they may still be related to the clinical score outcome. The model chose to diminish their weights because they bring no additional information in comparison to the other speech features.

## Discussion

Our multicentered prospective study aimed at predicting the clinical scores of different visits of 103 individuals carrying the mutant Htt gene leading to Huntington’s disease, using machine learning analyses of speech productions. We used speech features extracted from forward and backward counting—a task that lasts less than 40 s, even in patients at an advanced stage. We showed that measures of speech production accurately predict the clinical measures in HD, within the 12% to 20% range for the functional, motor, and cognitive, and composite cUHDRS (The Mean Absolute Error is divided by the maximum observed range to obtain these values). Speech features improved predictions from demographics and genetics characteristics alone by around 17% in relative terms. In particular, the predicted cUHDRS had an equivalent inter-rater agreement score (ICC) in the “good” reliability range. Finally, the Mean Duration and Standard Deviation of Durations of Silences correlated significantly with the atrophy of the striatum.

These results may lead to the construction of reliable, discriminative and applicable diagnostic tools for the prediction of the progress of the symptoms. Our forward/backward counting task provides a good compromise between the different requirements for a usable language-based battery in a clinical setting: accuracy (to measure the evolution of the condition), ease of use, and multidimensionality (capability for one single marker to capture several dimensions of the disease [5]).

As for accuracy, for machine learning systems to be clinically valuable, assessing only the statistical significance of the group performance (here the Cohort Mean Performance) is insufficient [7]. The derived scores should be predictive enough at the individual level to be used for clinical decision making. This is why, to assess their accuracy, we compared our predicted scores with standard tests performed by neurologists [12]. As expected, the ICCs from machine learning models did not match the ones of expert clinical raters [3, 4, 49]. However, their capacity to assess the patients frequently could reduce the cost to evaluate clinical therapies in HD by increasing the measures of an individual, thus permitting the reduction of the required number of participants in clinical trials [50].

As for ease of use, the forward and backward counting does not require the involvement of any expert nor training for patients’ recording. This constitutes a major progress considering that despite its worldwide dissemination and its excellent acceptability, the interrater reliability of the UHDRS between neurologists decreases in absence of annual certification [3]. Audio data can be collected over the phone, allowing not only remote but also out of sync assessments between health professionals and patients [51]. The limited vocabulary and deterministic sequences expected from participants allows easier development of fully automated procedures potentially reducing further annotation time. In contrast, other batteries like the Cantab [52], and the HD-CAB [6], require longer assessments, are not easy to administer and cannot currently be performed remotely.

Finally, as regards multidimensionality, our simple speech test, allows measuring, on the top of language, the different components of the UHDRS (cognitive, motor and functional).

Our results are consistent with previous ones in HD concerning the different dimensions that are affected during spoken language production. Our 60 speech features coded articulatory and phonatory deficiencies, rhythm and temporal statistics, and added seldom studied collateral track additions, sequence and perseverations. We showed that rhythmic and articulatory features were particularly sensitive to the progress of the disease. Rhythmic features well reflected motor and cognitive disabilities (Fig. 5) and correlated the most with the striatal volume (Table 3). This latter result is consistent with Hinzen’s findings on a storytelling task in which the composite quantitative score capturing the rhythm was the only one correlated with striatal atrophy. This confirms the involvement of the striatum in motor programs of phones and syllables, and their sequential structure and timing[14] Besides, we also found that articulatory features were linked to various HD deficits (global, motor, functional but not as much to the cognitive scores: SDMT, Stroop Word and Stroop Interference) like in previous reading tasks [18, 27, 29] and storytelling tasks [26].

We obtained robust estimations of clinical scores, even though using a relatively simple task. Yet, the strength of rhythmic and phonatory impairments in HD has been shown to depend on the cognitive load of the task used to elicit speech. Vogel and authors studied the speech disturbances of manifest and premanifest mutant Htt gene carriers while performing a spectrum of tasks from low to high cognitive load [27]. In their study, rhythmic deficits correlated with the TMS only when measured from a reading task (Percentage of silence *R* = 0.4) and a monologue task (Percentage of silence *R* = 0.5) but not from automated speech (recitation of the days of the week, Percentage of silence *R* = 0.08). Similarly, although HD participants have difficulties to sustain the vowel /a/ steadily for a few seconds compared to premanifest patients [30], speech features extracted from this simple task could not improve the clinical score extracted from demographics alone [38]. In our present study, we used both an automatic task (counting forward) and a more cognitive complex task (counting backward). A post hoc analysis shows that the forward counting task alone, which involves an automatic sequence yields lower predictions than the backward counting sequence. As described in the methods, when participants perform the backward counting, they need to inhibit the automatic number forward recitation and disengage from the overlearned forward sequence of numbers just previously performed. In addition, we used a dual task (of holding hands and closing eyes) [53] which is known to increase reaction times and errors [54]. As seen in Fig. 5, perseveration features are more salient in the backward compared to the forward test confirming the importance of cognitive load when estimating the symptoms of HD participants.

Here, we focused on the measurement of rather low-level acoustic features in a rather simple task for its potential for automation and applicability in different languages with minimal adaptation. Other studies have demonstrated that HD symptoms also include higher levels of language processing (conceptual, lexical, syntactic planning) [26]. Adding such high level features could improve the accuracy of a test battery over low level speech features. However, it was shown [55] that the extraction of high level features from 10 min of speech imposed two hours of annotation by experts including the identification of “Who speaks when?”, “What is said?”, and “How is it said?”. Current Artificial Intelligence (AI) research is being done to replace the expert linguist by automatic systems in order to reduce the cost of analyzing such tests. an automatic speech recognition system that could recognize the words was built [29] (“What is said?”) directly from audio recordings of the ‘GrandFather Passage’ story yielding to 85% accuracy when classifying HD from controls using speech features (speech rate, pauses, fillers, and goodness of pronunciation). However, humans were still required to segment manually the turns between doctor and patient, and the boundaries between sentences before feeding the automatic transcriber. Surprisingly, “Who speaks when” is still more challenging for algorithms than for humans when the audio comes from naturalistic and clinical settings (see the low performance in engineering DIHARD challenges [56]). Even when using state-of-the-art models, the reliability of “Who speaks when” in a clinical context remains too low for clinical use [38]. More powerful models and larger datasets will eventually overcome these limitations. The combination of different objective sources is an opportunity to increase the predictive power of the clinical scores based on speech features. In future work, this would be of great interest to combine speech features to other objective measures such as the Q-motor [57]. Yet, this still represents a technical challenge as the number of dimensions to analyze increase.

Our study presents some limitations that could be overcome in future works. The number of participants limited to a hundred here might impact the generalization results. Focusing on French gene-carriers of the mutant Htt gene should not constitute a problem, the analysis of results from five languages in Parkinson’s disease was found equivalent [58]. Our task was designed with as much as language-independent features, but it does not warrant the generalization of our results across languages and centers. Despite Huntington's disease combining the major features of NDDs—motor, psychiatric and cognitive disorders, the dissemination of our method requires validation in each individual disease of interest.

In conclusion, this is the first machine learning model combined with speech study that reliably estimated the scores of classical scales assessing several domains for pre-HD individuals and HD participants. One of its strengths is that the reliability of the predictive models closely match the observed data from neurologists and neuropsychologists for HD, without any ambiguity on the reliability of the data as methods were pre-registered before analyses. Being able to evaluate the severity of the different symptoms so quickly and potentially remotely has both clinical and experimental relevance in HD. This will likely reduce the human and financial burden for the follow-up of patients and help to reduce the cost of future disease modifying therapeutic trials.
